# Supplementary figures and images for: Decipher the complexity of cis-regulatory regions by a modified Cas9
Source: PLoS One. 2020 Jul 2;15(7):e0235530. doi: 10.1371/journal.pone.0235530 (PMC7332081; doi:10.1371/journal.pone.0235530)

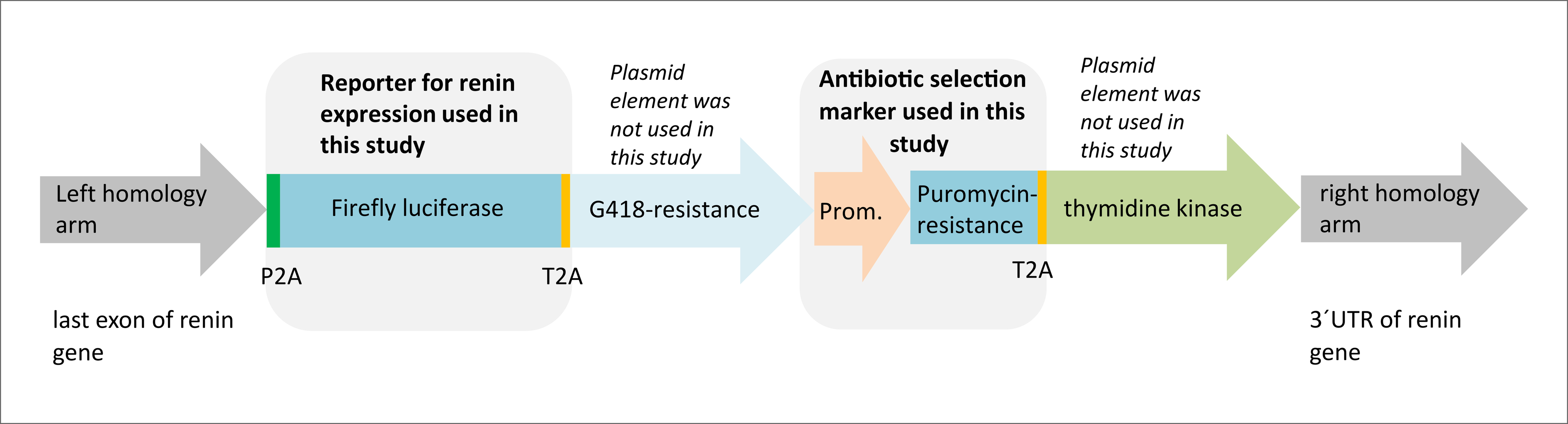

Supplement: S1 Fig — The cassette contains the Firefly luciferase, which was used as a reporter for REN expression. Puromycin was used to select the cells. The homology arms were required for the correct in frame insertion of the cassette. The elements G418-resistance and thymidine-kinase also contained in the cassette were not used in this study. (TIF) [file pone.0235530.s001.tif]

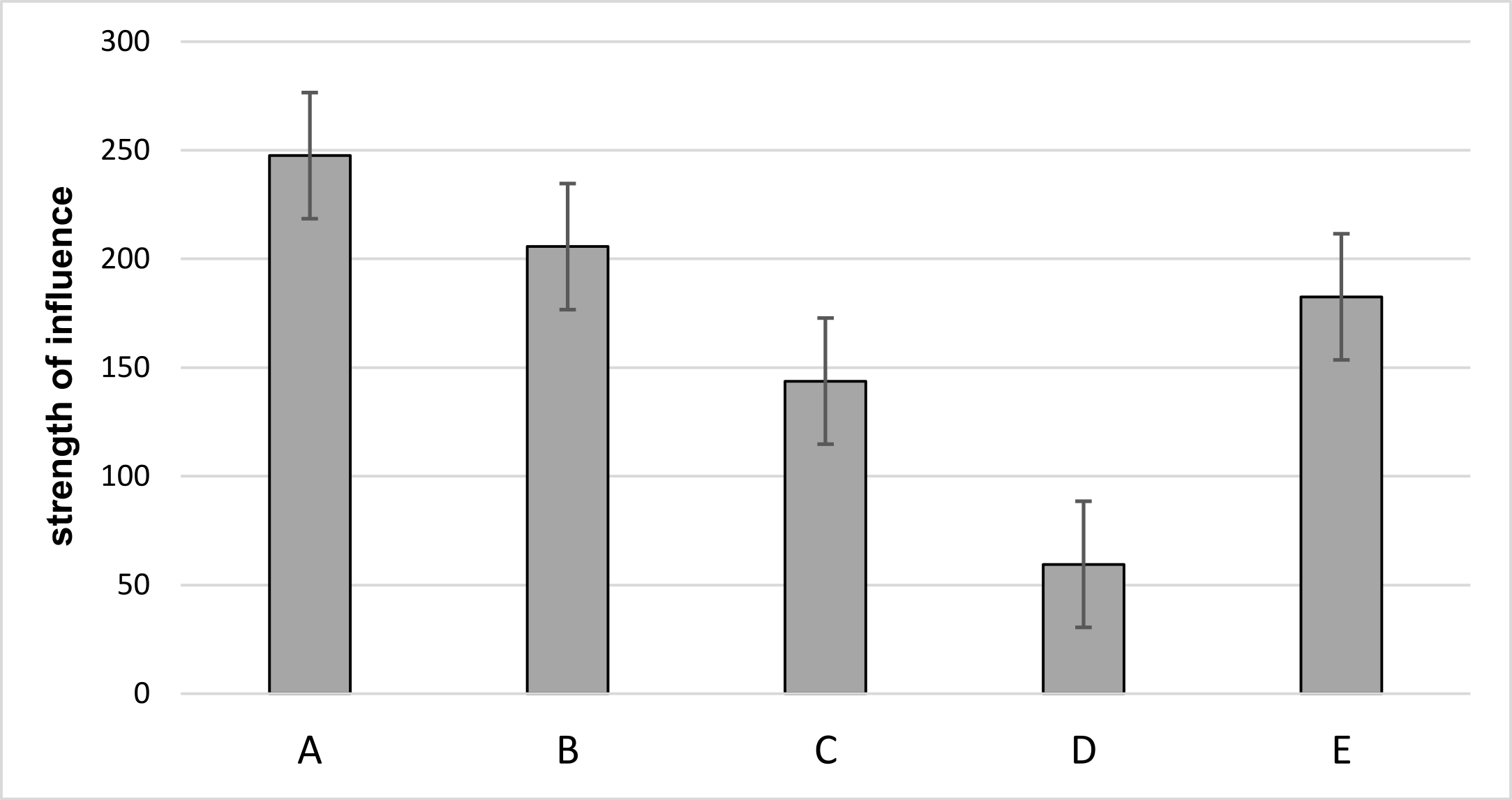

Supplement: S2 Fig — All investigated sequences significantly affect the activation of REN. Considering the sum model, guide A reached the most potent effect. Significances resulted from the linear regression modelling with the lm function of the stats package in R. (p-values: *<0,05, ***<0,0005). (TIF) [file pone.0235530.s002.tif]
