# Supplementary material for: Decipher the complexity of cis-regulatory regions by a modified Cas9
Source: PLoS One. 2020 Jul 2;15(7):e0235530. doi: 10.1371/journal.pone.0235530 (PMC7332081; doi:10.1371/journal.pone.0235530)
Supplement: S1 Table — (DOCX) [file pone.0235530.s003.docx]

**S1 Table. Luciferase activity for all 32 guide combinations at three different time points.**

|  | **Time point 36.2 hours** | | | **Time point 48 hours** | | | **Time point 60.5 hours** | | | |
| --- | --- | --- | --- | --- | --- | --- | --- | --- | --- | --- |
| **Guide combinations** | **Mean** | **SD** | **SD/**  **Mean** | **Mean** | **SD** | **SD/**  **Mean** | **Mean** | **SD** | **SD/**  **Mean** |  |
| **_____** | 49.0 | 3.0 | 0.06 | 71.7 | 17.6 | 0.25 | 91.3 | 19.7 | 0.22 |  |
| **____E** | 107.0 | 21.7 | 0.20 | 235.0 | 56.7 | 0.24 | 641.3 | 170.2 | 0.27 |  |
| **___D_** | 177.3 | 24.2 | 0.14 | 551.7 | 74.2 | 0.13 | 1530.7 | 265.0 | 0.17 |  |
| **___DE** | 387.0 | 67.7 | 0.18 | 1202.0 | 218.8 | 0.18 | 3654.0 | 692.2 | 0.19 |  |
| **__C__** | 187.0 | 25.4 | 0.14 | 507.0 | 103.5 | 0.20 | 1449.3 | 410.5 | 0.28 |  |
| **__C_E** | 592.3 | 119.7 | 0.20 | 1751.7 | 501.7 | 0.29 | 5094.3 | 1433.2 | 0.28 |  |
| **__CD_** | 301.3 | 25.2 | 0.08 | 828.7 | 276.7 | 0.33 | 2259.0 | 640.9 | 0.28 |  |
| **__CDE** | 550.7 | 31.2 | 0.06 | 1353.0 | 355.3 | 0.26 | 3537.0 | 831.3 | 0.24 |  |
| **_B___** | 557.0 | 91.8 | 0.16 | 1421.7 | 221.6 | 0.16 | 3096.7 | 317.2 | 0.10 |  |
| **_B__E** | 2726.0 | 360.2 | 0.13 | 6688.3 | 1303.9 | 0.19 | 15788.0 | 2150.1 | 0.14 |  |
| **_B_D_** | 1428.7 | 168.1 | 0.12 | 3592.0 | 818.1 | 0.23 | 8922.0 | 1609.1 | 0.18 |  |
| **_B_DE** | 2666.3 | 545.0 | 0.20 | 6820.7 | 1285.9 | 0.19 | 16513.3 | 2448.4 | 0.15 |  |
| **_BC__** | 1788.3 | 336.7 | 0.19 | 4589.3 | 1083.5 | 0.24 | 11759.3 | 2247.7 | 0.19 |  |
| **_BC_E** | 2705.7 | 489.0 | 0.18 | 6018.3 | 449.9 | 0.07 | 15614.3 | 1783.2 | 0.11 |  |
| **_BCD_** | 2137.0 | 404.7 | 0.19 | 4939.0 | 1300.3 | 0.26 | 11592.0 | 2054.3 | 0.18 |  |
| **_BCDE** | 2171.7 | 196.2 | 0.09 | 4267.3 | 427.9 | 0.10 | 10031.0 | 1240.1 | 0.12 |  |
| **A____** | 284.7 | 36.6 | 0.13 | 808.5 | 62.9 | 0.08 | 1762.5 | 115.3 | 0.07 |  |
| **A___E** | 1856.3 | 452.6 | 0.24 | 5416.5 | 68.6 | 0.01 | 12770.0 | 499.2 | 0.04 |  |
| **A__D_** | 1991.0 | 161.6 | 0.08 | 6531.0 | 598.2 | 0.09 | 15846.0 | 1077.6 | 0.07 |  |
| **A__DE** | 2469.7 | 172.4 | 0.07 | 8108.0 | 99.0 | 0.01 | 19472.5 | 379.7 | 0.02 |  |
| **A_C__** | 2211.7 | 439.0 | 0.20 | 6760.0 | 52.3 | 0.01 | 16231.0 | 849.9 | 0.05 |  |
| **A_C_E** | 3385.7 | 269.2 | 0.08 | 9675.0 | 355.0 | 0.04 | 23013.0 | 2050.6 | 0.09 |  |
| **A_CD_** | 2460.7 | 204.9 | 0.08 | 7018.5 | 236.9 | 0.03 | 15940.5 | 466.0 | 0.03 |  |
| **A_CDE** | 2463.0 | 172.0 | 0.07 | 5929.5 | 842.2 | 0.14 | 13497.5 | 1257.9 | 0.09 |  |
| **AB___** | 659.7 | 41.8 | 0.06 | 1365.0 | 95.5 | 0.07 | 2972.7 | 167.9 | 0.06 |  |
| **AB__E** | 2202.7 | 271.1 | 0.12 | 5122.3 | 464.3 | 0.09 | 11916.3 | 914.0 | 0.08 |  |
| **AB_D_** | 1715.7 | 225.0 | 0.13 | 3983.0 | 289.3 | 0.07 | 9275.7 | 971.3 | 0.10 |  |
| **AB_DE** | 2556.7 | 301.8 | 0.12 | 5555.3 | 243.6 | 0.04 | 13489.7 | 1206.6 | 0.09 |  |
| **ABC__** | 2218.7 | 121.1 | 0.05 | 4600.7 | 467.0 | 0.10 | 10911.3 | 1179.5 | 0.11 |  |
| **ABC_E** | 3182.0 | 409.5 | 0.13 | 6462.7 | 1123.7 | 0.17 | 15028.3 | 1855.3 | 0.12 |  |
| **ABCD_** | 2197.7 | 173.2 | 0.08 | 4471.7 | 400.2 | 0.09 | 10278.7 | 1222.7 | 0.12 |  |
| **ABCDE** | 2065.3 | 248.1 | 0.12 | 3965.7 | 688.0 | 0.17 | 9129.0 | 1615.1 | 0.18 |  |

*REN* activation represented as means and standard deviations (SD) of luciferase activity from all 32 guide combinations at three different time points. The given hours information of the time points is rounded. Each experiment n=6.
