# Supplementary material for: Decipher the complexity of cis-regulatory regions by a modified Cas9
Source: PLoS One. 2020 Jul 2;15(7):e0235530. doi: 10.1371/journal.pone.0235530 (PMC7332081; doi:10.1371/journal.pone.0235530)
Supplement: S1 Script — (PDF) [file pone.0235530.s004.pdf]

S3 – R script source code

```
# path to data
setwd("C:/…")
# please make sure that this library ggplot2 is installed
library(ggplot2)

# get rid of all potential old data
## detach(list = ls()) # activate if neccessary
## rm(list = ls()) # activate if necessary

jj <-
  read.table(file = "Transfektionen_Zuordnung_v005_DatRaw_filtered.csv", header =
               TRUE, sep = ";") # load data file

# this timepoint was used to calculate the models,
# appr. 60 hours after transfection
jjj <-
  subset(jj, cycle == 430)

# just check for data structure
dim(jjj)
attach(jjj)

######### random control model #########

AA <- sample(A)
BB <- sample(B)
CC <- sample(C)
DD <- sample(D)
EE <- sample(E)

lmMcontrol <- lm(cps ~ AA + BB + CC + DD + EE)
AIC(lmMcontrol)
summary(lmMcontrol)

########### model definition ############
# assignement of sum model
lmMsum <- lm(cps ~ A + B + C + D + E)
# assignement of interaction model
lmMinteraction <- lm(cps ~ A * C * D * E * B)

########### model characterization ######
AIC(lmMsum)
AIC(lmMinteraction)
summary(lmMsum)
summary(lmMinteraction)

########### error analysis #########
# calculation of absolute prediction error
fehler <-  abs(predict(lmMsum) - cps)
zuordnung <- (rep("sum model", 192))
fehlerdata <- data.frame(fehler, zuordnung)

# calculation of absolute prediction error
fehler <-  abs(predict(lmMinteraction) - cps)
zuordnung <- (rep("interaction model", 192))
fehlerdata2 <- data.frame(fehler, zuordnung)

# independent 2-group Mann-Whitney U Test
#wilcox.test(y,x) # where y and x are numeric
wilcox.test(fehlerdata$fehler, fehlerdata2$fehler)
fehlerdata <- rbind(fehlerdata, fehlerdata2)

ggplot(data = fehlerdata, aes(x = zuordnung, y = fehler))  + geom_boxplot()
detach(jjj)
```
